# Correlation between microtubule-associated gene expression and chemosensitivity of patients with stage II non-small cell lung cancer

**DOI:** 10.3892/etm.2013.1007

**Published:** 2013-03-14

**Authors:** HONG JIANG, XIN-MING YU, XING-MING ZHOU, XIAO-HONG WANG, DAN SU

**Affiliations:** 1 Department of Thoracic Surgery, Key Laboratory of Thoracic Tumor Diagnosis (Esophagus, Lung) and Treatment Technology Research, Zhejiang Cancer Hospital, Hangzhou, Zhejiang 310022, P.R. China;; 2 Department of Chemotherapy, Zhejiang Cancer Hospital, Hangzhou, Zhejiang 310022, P.R. China; 3 Cancer Institute, Zhejiang Cancer Hospital, Hangzhou, Zhejiang 310022, P.R. China

**Keywords:** lung tumor, chemotherapy, β-tubulin-III, stathmin

## Abstract

The aim of this study was to explore the correlation between mRNA expression of β-tubulin-III and stathmin in patients with stage II non-small cell lung cancer (NSCLC) and the chemosensitivity to Navelbine plus cisplatin (NP), as well as to provide a basis for personalized treatment. A single-gene quantitative test was performed to detect the mRNA expression of β-tubulin-III and stathmin in the tumor tissue of patients with stage II NSCLC. All the patients underwent NP treatment following surgery and were followed-up to record their disease-free survival (DFS) and overall survival (OS). Statistical analyses were conducted to investigate the correlation between β-tubulin-III and stathmin mRNA expression and DFS and OS in the patients. β-tubulin-III mRNA expression was associated with OS in the 73 patients (P=0.003) and DFS was correlated with β-tubulin-III mRNA expression and lymphatic metastasis (P<0.01). Stathmin mRNA expression was not correlated with OS or DFS (P>0.05). OS and DFS were longer in the patients with low β-tubulin-III mRNA expression than in those with high β-tubulin-III mRNA expression (P<0.01); there was no significant change in OS and DFS between the patients with high and low mRNA expression of stathmin (P>0.05). The mRNA expression levels of β-tubulin-III in the tumor tissue of patients with stage II NSCLC may be considered as an index of prognosis and chemosensitivity, as well as a reference for personalized chemotherapeutic applications in patients.

## Introduction

Numerous clinical studies have demonstrated that postoperative chemotherapy prolongs the survival of patients with non-small cell lung cancer (NSCLC). The 2011 guidelines from the National Comprehensive Cancer Network (NCCN) regarding NSCLC diagnosis and treatment state that the standard treatment for patients with a good performance status scale (PS) was a platinum-based double drug combination (platinum combined with vinblastine, paclitaxel or gemcitabine). However, NSCLC with the same pathology and stage exhibits considerable heterogeneity in its sensitivity to chemotherapy. Currently, individual treatment of cancer is performed, which requires distinguishing the NSCLC to enable the treatment to be matched with the pathology and stage. Specific tumor markers are considered a good method for identifying changes in an individual patient and the joint detection of a series of tumor molecular markers aids the chemotherapeutic efficacy.

Studies have demonstrated that tubulin is a target site for a number of antitumor drugs, among which β-tubulin has seven species of isomers and its coding genes are located in 6p21.3 (1). According to the combined detection with isoelectric focusing and mass spectrometry, β-tubulin-III has been recognized as the only marker with a resistance to a microtubule drug among the seven species of isomers. A clinical study confirmed that β-tubulin-III expression is associated with chemotherapeutic resistance to the microtubule-binding protein; however, the conclusions are inconsistent and occasionally opposing (2). Stathmin, an important microtubule depolymerizing protein, plays a crucial role as a cell signal transduction molecule in cell differentiation, tissue regeneration and restoration, particularly in tumor initiation, development and phenotype determination. Effective inhibition of stathmin expression in malignant tumors suppresses tumor cell division and proliferation, and also promotes cell apoptosis and collaborates with anti-microtubule drugs to produce an antitumor effect (3). Stathmin is likely to be a promising therapeutic target in tumor treatment (4,5).

In the current study, a total of 73 patients with stage II NSCLC who underwent surgery were selected and recieved postoperative chemotherapy of Navelbine plus cisplatin (NP). The mRNA expression levels of β-tubulin-III and stathmin were detected in tumor tissue and the disease-free survival (DFS) and overall survival (OS) rates of the patients were recorded. Statistical analysis was performed to explore the correlation between gene expression levels and DFS and OS and to investigate the correlation between β-tubulin-III and stathmin mRNA expression with chemotherapeutic efficacy, to provide a basis for the establishment of personalized therapy.

## Materials and methods

### Clinical samples

All subjects were screened from the specimen database of The Zhejiang Cancer Hospital. This study was conducted in accordance with the Declaration of Helsinki and with approval from the Ethics Committee of Zhejiang Cancer Hospital. Written informed consent was obtained from all participants. Following surgery, the pathology of NSCLC was classified according to the tumor, lymph nodes and metastasis (TNM) staging system of the 2009 Union for International Cancer Control (UICC), through which we selected 73 NSCLC patients who underwent radical surgery to remove a tumor between September 2007 and June 2010. Two 0.5 cm^3^ sections of tumor tissue were collected and processed into paraffin sections for measurement of β-tubulin-III and stathmin mRNA expression levels. The clinical features of the patients were as follows: age ≤60 years in 55 cases, >60 years in 18 cases; tumor size ≤3 cm in 22 cases, >3 cm in 51 cases; no lymphatic metastasis in 28 cases, lymphatic metastasis in 45 cases; high-moderate differentiation in 53 cases, low differentiation in 20 cases; adenocarcinoma in 33 cases, squamous cell carcinoma in 36 cases and other pathological types in 4 cases.

### Adjuvant chemotherapy

The patients underwent postoperative NP chemotherapy; 75 mg/m^2^ cisplatin was administered for 3 days and 25 mg/m^2^ Navelbine was administered on days 1 and 8. This treatment was repeated every 3 weeks. Only one patient recieved two cycles of chemotherapy due to bone metastasis and all other patients finished 3–4 cycles of chemotherapy in our hospital.

### DFS and OS collection

All patients were followed-up by phone by specified doctors and received a call every 3 months. The information was collected from 70 patients with a follow-up rate of 95.89%; the loss of follow-up was processed as the censored value.

### mRNA detection

A QuantiGene assay was performed by Gene Technology (Shanghai) Co., Ltd. to detect mRNA expression levels using an Affymetrix QuantiGene Plex 2.0 assay (Panomics, Fremont, CA, USA). The protocol was as follows: 3–5 pieces of paraffin sections were lysed and completely digested by lysis buffer. After centrifuging at 16,000 × g at room temperature, the supernatant was collected and mixed with microbeads and probes, followed by incubation at 54°C at 600 rpm overnight. Then, all the substance that had not hybridized to the microbeads was washed away in a magnetic field. Cascade amplification was performed on the captured RNA signals by bDNA technology. During this process, including pre-amplification and amplification, the samples were incubated with labeled probes for the two steps at 50°C at 600 rpm for 1 h,. The samples were washed three times using washing buffer following each incubation. Finally, gene expression data was recorded following streptavidin, phycoerythrin conjugated (SAPE) labeling by Luminex 200 (Luminex Corporation, Austin, TX, USA). Phycoerythrin is a protein that produces a bright red-orange fluorescence. Our improved phycoerythrin streptavidin (SA-5207) is substantially brighter than previous conjugates. Phycoerythrin streptavidin may be used on gene chips, in cell sorting and in tissue staining. The detailed protocol was according to the method described by Eastham *et al*(6). The hypoxanthine phosphoribosyltransferase 1 (HPRT1) gene was set as the quality control gene. Therefore, the copy number of the target gene together with the HPRT1 gene was assessed in each sample. The median value was considered as the boundary between the positive and negative.

### Statistical analysis

Univariate analysis was conducted using the COX model. The survival curve was drawn using the Kaplan-Meier method and checked by the Log-rank test using SPSS 17.0 software (SPSS Inc., Chicago, IL, USA). P<0.05 was considered to indicate a statistically significant result.

## Results

### β-tubulin-III mRNA expression

The mRNA expression levels of β-tubulin-III in the patients were as follows: mean, 1.250011; median, 0.939473; minimum, 0.2893; maximum, 4.9174; and variance, 1.018.

### Stathmin mRNA expression

The mRNA expression of stathmin in the patients were as follows: mean, 0.916739; median, 0.6318; minimum, 0.335; maximum, 5.0746; and variance, 0.975.

### Follow-up time

**Figure 1:**
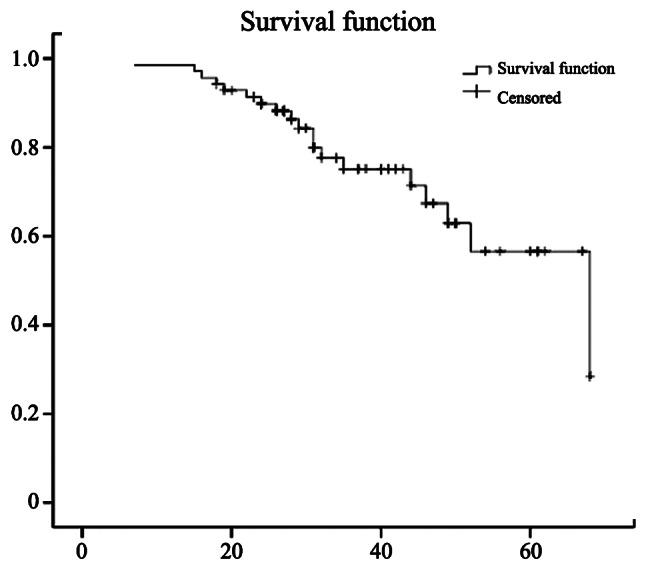
Survival curve of 70 patients.

The follow-up time was between January 17, 2007 and May 31, 2012 and the median follow-up time was 48 months. In 70 cases, 23 patients relapsed and had metastasis and 19 patients succumbed during follow-up. The 3- and 5-year survival rates were 75.1 and 56.6%, respectively. The median survival time was 68 months (95% CI, 45.21–90.79 months). The survival curve of the patients is shown in Fig. 1.

### Univariate analysis

Univariate analysis of OS and DFS was performed with the COX model, which indicated that the OS of patients was correlated with β-tubulin-III expression and DFS was associated with β-tubulin-III expression and lymphatic metastasis. However, the OS and DFS of patients were not correlated with the expression level of stathmin, age, tumor pathological type, tumor size or degree of differentiation (Tables I and II).

### General information

**Figure 2:**
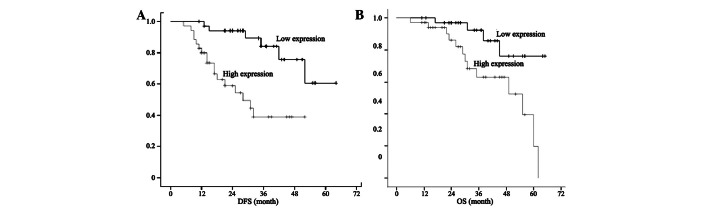
(A) DFS of patients with different β-tubulin-III expression levels (χ^2^=5.693, P=0.015). (B) OS of patients with different β-tubulin-III expression levels (χ^2^=8.369, P=0.004). DFS, disease-free survival; OS, overall suvival.

Five patients with negative β-tubulin-III expression had a relapse and metastasis and two of these patients succumbed during follow-up. Eighteen patients with positive β-tubulin-III expression had a relapse and metastasis and 17 of these patients succumbed during follow-up. The Kaplan-Meier method was utilized to produce a survival curve, which revealed that the OS and DFS were longer in patients with low β-tubulin-III mRNA expression levels compared with those with high β-tubulin-III mRNA expression levels (Fig. 2).

**Figure 3:**
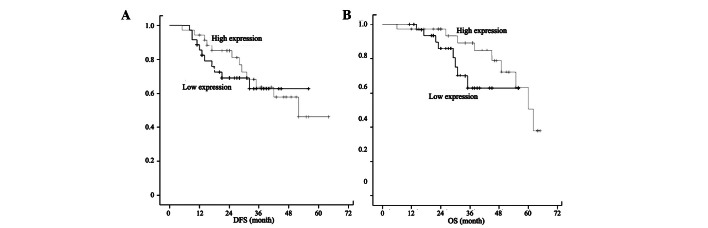
(A) DFS of patients with different stathmin expression levels (χ^2^=0.227, P=0.633). (B) OS of patients with different stathmin expression levels (χ^2^=1.907, P=0.167). DFS, disease-free survival; OS, overall survival.

Fourteen patients with negative stathmin expression had a relapse and metastasis and 11 of these patients succumbed during follow-up. Nine patients with positive stathmin expression had a relapse and metastasis and 6 of these patients succumbed during follow-up. The Kaplan-Meier method was utilized to produce a survival curve, which revealed no significant change in the OS and DFS between patients with low and high stathmin mRNA expression levels (Fig. 3).

## Discussion

The theme of the 2009 American Society of Clinical Oncology (ASCO) annual meeting was ‘personalized tumor treatment enables the cancer patients to live longer and better’, which should be considered when treating NSCLC patients. The aim of personalized therapy is to treat each cancer patient according to the biological features of the tumor and pharmacogenomics. In 2006, a landmark article on personalized chemotherapy for lung cancer was published in the New England Journal of Medicine, which indicated that only patients with low expression of excision repair cross complementation 1 (ERCC1) benefit from adjuvant chemotherapy (7). Hereafter, personalized chemotherapy based on pharmacogenomics, which draws great attention among the medical community, as well as the factors for chemosensitivity prediction and prognosis have become the focus. The expression of several factors, including the 5′-endonuclease of ERCC1, ribonucleotide reductase subunit M1 (RRM1) and breast cancer susceptibility gene 1 (BRCA1), are closely correlated with chemotherapeutic efficacy and prognosis, and have become crucial factors for predicting the efficacy of personalized chemotherapy (8–11).

Previous studies demonstrated that β-tubulin-III has a close correlation with the sensitivity to chemotherapeutics, which act on microtubules. A number of studies have confirmed that an increased concentration of β-tubulin-III is associated with decreased sensitivity to paclitaxel drugs (12,13).

Our study demonstrated that among patients with stage II NSCLC who underwent NP therapy following surgery, the OS and DFS were longer in patients with low β-tubulin-III mRNA expression levels than in patients with high β-tubulin-III mRNA expression levels. β-tubulin-III expression is considered an index for predicting the chemosensitivity to NP therapy and β-tubulin-III mRNA expression is recognized as an independent index for the prognosis of patients. However, the results of studies worldwide are inconsistent and occasionally opposing. Séve *et al* selected stage IB-II NSCLC patients and identified that patients with high β-tubulin-III expression levels have a shorter progression-free survival (PFS) and OS compared with those with low β-tubulin-III expression levels among patients who underwent surgery. However, the situation was the opposite among patients that underwent surgery and adjuvant NP therapy; the PFS and OS in the patients with high β-tubulin-III expression were longer than in those with low β-tubulin-III expression levels (14). One study performed immunohistochemistry on stage III–IV NSCLC to detect β-tubulin-III expression in the tumor tissue, which revealed that the expression level of β-tubulin-III was not correlated with the response rate of vinblastine therapy; however, it was associated with disease progression time. The patients with high β-tubulin-III expression had shorter PFS and OS than those with low β-tubulin-III expression, which indicates that patients with high β-tubulin-III expression levels have a vinblastine tolerance (15). Reiman *et al* investigated the correlation between β-tubulin-III expression and prognosis of 1,149 patients in four clinical trials whose NSCLC had been resected. The authors identified that β-tubulin-III expression level may be used as a prognostic index but not as an index for predicting chemotherapy efficacy (16). A number of studies have produce inconsistent results. This may be a result of varying predictions of β-tubulin-III expression for the early and late stages of NSCLC. Furthermore, the prediction of Navelbine efficacy through β-tubulin-III expression was worse than that of paclitaxel, which required numerous prospective trials to confirm (17).

Stathmin, an unstable microtubule protein, plays a crucial role in modulating the dynamic equilibrium of the cell micro-tubule system through phosphorylation and dephosphorylation during various stages of the cell cycle. Studies on several types of lung cancer cells have shown that microtubule polymerization has an impact on its binding to chemotherapeutic agents. The enhanced microtubule polymerization increases the binding of microtubules to paclitaxel instead of vinblastine, affecting the response to chemotherapeutics in patients with lung cancer (18,19).

The current study demonstrated that, following surgery, stathmin expression in patients with stage II NSCLC is not correlated with patient prognosis and should not be used as an independent predictor for prognosis. There was no significant change in the OS and DFS between patients with high and low mRNA expression levels of stathmin. Pu *et al* reported that the expression level of stathmin is negatively correlated with the efficacy of NP therapy in late NSCLC, indicated by immunohistochemistry (20), which is inconsistent with our results. One explanation is that stathmin expression, as well as β-tubulin-III, predicts differently for the early and late stages of NSCLC. Stathmin, modulating the dynamic equilibrium of the cell microtubule system through phosphorylation and dephosphorylation during various stages of the cell cycle, has a different function at different stages of the cell cycle. The stathmin protein: promotes microtubule depolymerization during mitosis interphase; it phosphorylates and the spindle apparatus forms during the early phase of mitosis; and it dephosphorylates and the spindle apparatus depolymerizes during the late phase of mitosis; this process then repeats (21). The second explanation is that the samples, methods and standards for detection are different. At present, most studies focus on late NSCLC, utilizing a puncture or bronchofiberscope technique to collect specimens. The qualitative methods of PCR and immunohistochemistry are commonly applied; however, quantitative detection is rarely performed. Moreover, limited samples and the length of follow-up time may also result in differences. All these factors may account for the inconsistent results. Therefore, we are using more methods and collecting a larger number of samples to assess the gene expression levels in tumor tissue in order to achieve more accurate results.

## Figures and Tables

**Table I:** Univariate COX analysis of patient overall survival.

							95% CI applied to Exp(B)	
Parameter	B-value	SE	Wald	df	Sig.	Exp(B)	Lowest	Highest
Gender	−0.606	0.653	0.860	1	0.354	0.546	0.152	1.963
Age	0.564	0.400	1.991	1	0.158	1.758	0.803	3.847
TUBB3	0.402	0.178	9.032	1	0.003	1.548	1.152	1.992
STMN	−0.014	0.242	0.003	1	0.953	0.986	0.613	1.586
Pathology	−0.049	0.401	0.015	1	0.902	0.952	0.434	2.090
Size	0.409	0.301	1.848	1	0.174	1.506	0.835	2.716
Differentiation	0.999	1.038	0.926	1	0.336	2.716	0.355	20.779
LNM	0.216	0.158	1.855	1	0.173	1.241	0.910	1.693

SE, standard error; CI, confidence interval; TUBB3, β-tubulin-III; STMN, stathmin; LNM, lymph node metastasis.

**Table II:** Univariate COX analysis of patient disease-free survival.

							95% CI applied to Exp(B)	
	B-value	SE	Wald	df	Sig.	Exp(B)	Lowest	Highest
Gender	−0.032	0.506	0.004	1	0.950	0.969	0.359	2.614
Age	0.399	0.317	1.585	1	0.208	1.491	0.801	2.775
TUBB3	0.423	0.140	9.097	1	0.003	1.526	1.160	2.009
STMN	0.165	0.188	0.772	1	0.380	1.180	0.816	1.706
Pathology	0.304	0.354	0.741	1	0.389	1.356	0.678	2.712
Size	0.123	0.218	0.316	1	0.574	1.131	0.737	1.735
Differentiation	1.038	0.741	1.962	1	0.161	2.823	0.661	12.062
LNM	0.345	0.120	8.191	1	0.004	1.412	1.115	1.788

SE, standard error; CI, confidence interval; TUBB3, β-tubulin-III; STMN, stathmin; LNM, lymph node metastasis.
